# Risky sexual behaviors and associated factors among university young adults in Zambezi, Namibia

**DOI:** 10.4102/hsag.v30i0.2725

**Published:** 2025-03-28

**Authors:** Sylvia K. Mahoto, Honoré K. Mitonga, Eddy S. Likando

**Affiliations:** 1 School of Nursing and Public Health, Faculty of Health Sciences and Veterinary Medicine, University of Namibia, Oshakati, Namibia; 2 Faculty of Technology, University of Portsmouth, Portsmouth, United Kingdom

**Keywords:** STIs, sexually transmitted infections, HIV, young adults, adolescents, risky sexual behaviours, universities, higher learning institutions, Zambezi region, Namibia

## Abstract

**Background:**

Risky sexual behaviours (RSBs) are prevalent among young adults, particularly university students, increasing their vulnerability to sexually transmitted infections (STIs) and unplanned pregnancies. While various studies have explored these behaviours in Namibia, limited research exists in the Zambezi region, presenting a gap that this study addresses.

**Aim:**

This study investigates the prevalence of RSBs, associated factors and the need for targeted interventions among university students in the Zambezi region.

**Setting:**

The study was conducted at the University of Namibia’s Katima Mulilo Campus in the Zambezi Region, Namibia.

**Methods:**

A descriptive cross-sectional survey was conducted among 231 undergraduate students at the University of Namibia (Katima Mulilo Campus). The survey assessed sexual behaviour, STI knowledge and condom use practices.

**Results:**

Over 61% of participants reported being sexually active, with many initiating sexual activities before the age of 18. Condom use was inconsistent, with 40.7% reporting not using condoms in the past year. Additionally, 15.6% had contracted an STI in the last year, with 6% not seeking medical treatment. Key factors associated with RSBs included marital status and substance use, such as tobacco.

**Conclusion:**

The findings reveal a concerning prevalence of RSBs among university students in the Zambezi region. This underscores the need for targeted interventions addressing social and behavioural determinants of sexual risk-taking.

**Contribution:**

This study emphasises the importance of developing culturally sensitive interventions that promote consistent condom use and improve sexual health education to reduce STI risk and adverse sexual health outcomes.

## Introduction

One of the significant behavioural issues that poses a public health concern among adolescents is their involvement in risky sexual behaviours (RSBs) (Wakasa et al. 2021). The term RSBs encompasses actions that heighten an individual’s vulnerability to sexually transmitted infections (STIs), HIV, unintended pregnancy and emotional or psychological distress (Tadesse & Yakob 2015; World Health Organization 2023). Some authors differentiate between RSBs (where one knowingly takes a risk with a partner infected with an STI) and sexual risk behaviour, where an individual may not be aware of the risks but engages in behaviours that can still lead to negative health outcomes. This study defines RSBs as actions that increase the likelihood of contracting STIs or experiencing unplanned pregnancies. Based on published research, RSBs include engaging in unprotected intercourse, having multiple sexual partners, participating in sexual activity under the influence of substances or having sex immediately after exposure to pornographic material (Urassa et al. 2008; Kalolo et al. 2019).

In 2015, the global population included approximately 1.8 billion adolescents and young people (Kusheta et al. 2019). In the sub-Saharan African region, nearly a quarter of the population consists of young people, and it is projected that this proportion will increase substantially by 2050 (World Health Organization 2023). This demographic shift highlights the urgent need for addressing public health challenges among adolescents, particularly those related to sexual health.

Various studies conducted in sub-Saharan Africa, including Namibia, have highlighted the significant incidence of STIs, including HIV, among young individuals. These studies also show that females exhibit a higher prevalence of STIs compared to males (Chinsembu et al. 2008; Jaspan et al. 2006; Seidu et al. 2021). However, there is a difference in help-seeking behaviour, with women generally more likely to access healthcare than men. Understanding the accessibility of sexual health services to young adults, including STI testing and treatment, is crucial for interventions aimed at reducing STI rates (Keller 2020).

Furthermore, involvement in RSBs at a young age is associated with various adverse sexual health consequences, such as a heightened risk of STIs, intimate partner violence and unplanned or adolescent pregnancies (Manu et al. 2022). Contributing factors to early sexual debut include not only permissive sexual behaviour but also instances of sexual abuse or violence, which may also shape later RSBs.

Over the past few decades, Africa has witnessed a shift towards more permissive sexual attitudes. This shift may be linked to urbanisation, the rise of social media and improved access to education, particularly for females (Kharono et al. 2022; Masanja et al. 2021). However, despite extensive research on sexual behaviours across Namibia, there is a notable gap in data specific to the Zambezi region. This lack of region-specific information presents challenges for health authorities and policymakers in devising targeted interventions that address the unique needs of adolescents and young adults in this area.

To address this gap, the current study aims to provide up-to-date data on the prevalence and determinants of RSBs among university students in the Zambezi region of Namibia. The study investigates factors such as sexual activity, condom use and STI knowledge, contributing to a better understanding of the sexual health landscape in this population. The results can inform regional health authorities, researchers and organisations to craft effective, culturally sensitive intervention strategies aimed at reducing risky sexual practices and improving sexual health outcomes.

## Research methods and design

### Study setting

The Zambezi region in northeastern Namibia has a population of 98 849 (2016), with 51% female and an annual growth rate of 1.3%. Approximately, 31% of the population resides in urban areas (Namibia Statistics Agency 2011). The study was conducted at the University of Namibia’s Katima Mulilo Campus, which enrols about 1200 undergraduate students, mostly aged 19 years – 30 years. To ensure inclusivity, participants aged 18 years – 35 years were targeted, as some students older than 30 years old are still pursuing their degrees. The inclusion of students aged 18 years–35 years addresses the broader participation criteria. While most students are 19 years – 30 years, the inclusion of those up to 35 years avoids excluding older students. The university’s diverse faculties and programmes may have influenced the study’s findings, as academic background could affect behaviours related to sexual activity and risk factors. This was not accounted for in the analysis and should be acknowledged as a limitation. Future research could explore correlations between fields of study and sexual behaviours.

### Study design, participants and sampling

An institution-based cross-sectional study was conducted between March and July 2023 at the University of Namibia’s Katima Mulilo Campus. Out of approximately 1200 enrolled undergraduate students aged 18 years – 35 years, a total of 231 students participated in the study. All students within this age range were invited to participate, and only those who provided informed consent were included in the final sample. The sample size of 231 participants was based on convenience sampling, considering the total student population and anticipated response rates. While the sample represents a portion of the overall student body, the convenience sampling method may limit the generalisability of the findings.

### Study instrument and data collection

Data collection employed an online, self-administered questionnaire in English, designed based on established theories of sexual behaviour and risk factors. Variables were selected from existing literature to capture key demographic, socioeconomic and behavioural aspects, such as sexual activity, condom use and substance consumption. The questionnaire underwent pretesting with a separate group of students to assess clarity and cultural relevance, leading to revisions before implementation. The pilot data were excluded from the final analysis. Google Forms was used for data collection, ensuring accessibility. Participants were recruited through email invitations sent by the university registrar, and consent was implied by survey completion. Reminders were sent periodically over the 4-month data collection period, although the extended duration may have contributed to a lower response rate. Data quality was maintained through daily checks by the principal investigator, data validation and cleaning processes to ensure completeness and accuracy.

### Data analysis

The collected data were thoroughly cleaned and cross-checked to ensure accuracy before analysis. Data coding and analysis were performed using the Statistical Package for Social Sciences (SPSS) version 29 (Corp 2023). Descriptive statistics, including frequencies and percentages, were used to summarise participants’ demographic and socioeconomic characteristics. For inferential analysis, binary logistic regression models were employed to assess the association between independent variables and RSBs. The independent variables included age, gender, marital status, residence (urban vs. rural), year of study, sexual behaviours (e.g. number of sexual partners and condom use) and substance use (alcohol and tobacco consumption). Risky sexual behaviour, the dependent variable, was defined based on factors such as multiple sexual partners and inconsistent condom use. Variables with a *p*-value of less than 0.05 were considered statistically significant. The findings were presented in tables to enhance clarity and facilitate interpretation (see Table 3).

### Ethical considerations

Ethical approval to conduct this study was obtained from the Research Ethics Committee (REC) at the University of Namibia (Reference no.: KMC0005). All participants were informed about the study’s main objectives and provided written consent before participating.

## Results

The study recruited a total of 231 undergraduate students. Females comprised a slight majority, with 128 participants (55.4%), compared to 103 males (44.6%). The participants’ ages ranged from 18 years to 35 years, with most (38.5% or 89 students) falling between 24 and 26 years old. Nearly three-quarters of the participants (74.5%, or 172 students) lived off-campus, with the largest group (28.1%, or 65 students) residing in the Katima Mulilo urban constituency (see Table 1).

**TABLE 1:** Socio-demographic characteristics of study participants.

Variables	Character	*n*	%
Gender	Female	12	55.4
	Male	103	44.6
Age (years)	18–20	56	24.2
	21–23	56	24.2
	24–26	89	38.5
	27–30	13	5.6
	31+	17	7.4
Year of study	1st year	59	25.5
	2nd year	69	29.9
	3rd year	48	20.8
	4th year	55	23.8
Current place of residence	On campus (hostel)	59	25.5
	Off-campus	172	74.5
Marital status	Single (not in a relationship)	85	36.8
	Single (in a relationship)	88	38.1
	Married	20	8.7
	Divorced	3	1.3
	Co-habitating	35	15.2
Where did you grow up?	Urban	86	37.2
	Rural	1	0.4
	Urban and rural	144	62.3
With whom did you grow up?	Biological mother and father	74	32.0
	Biological father	29	12.6
	Biological mother	65	28.1
	Other (extended family)	63	27.3
Area (Constituency)	Kabbe South	17	7.4
	Kabbe North	15	6.5
	Linyanti	29	12.6
	Judea Lyamboloma	22	9.5
	Sibbinda	20	8.7
	Katima Mulilo Urban	65	28.1
	Rural Katima Mulilo	17	7.0
	Kongola	13	5.6
	Other regions in Namibia	33	14.3

Over 61% of respondents (141 students) reported having ever engaged in sexual intercourse. Among sexually active participants, nearly 39% (90 students) reported that their first sexual experience occurred before the age of 18 years. A smaller group (21%, or 51 students) reported never having had sexual intercourse. Most sexually active participants (40.7%, or 94 students) indicated that their first sexual encounter took place during their secondary school years, followed by university years (11.7%, or 27 students). A smaller percentage (8.7%, or 20 students) reported their first sexual experience during primary school. The most frequently cited reason for engaging in sexual intercourse was personal desire (43%, or 101 students). Marriage was a motivator for 4% (24 students), while peer pressure was the least cited reason (5%, or 12 students). Of the sexually active participants, 32.5% (75 students) were female, and 28.6% (66 students) were male, with most falling between the ages of 24–26 years (22.1%, or 51 students). First-year students reported the highest level of sexual activity, with 16.9% (39 students) reporting sexual intercourse compared to other academic years (see Table 1).

The study also examined the nature of participants’ first sexual partners. Over 42% (97 students) reported their first sexual encounter with a boyfriend or girlfriend, while 10.8% (25 students) reported sex with a commercial sex worker. Less frequent encounters involved teachers or lecturers (4.8%, or 11 students), and a small percentage (1.7%, or four students) reported sex with a stranger. The number of sexual partners also varied, with 37.7% of participants reporting one to two partners, followed by 16% who reported three to five partners. A smaller group (7.4%) reported having six to nine partners.

Contraceptive use during the last sexual encounter varied. Condoms were the most common method (26.4%), followed by the withdrawal method (19%). However, 7.8% of participants reported not using any form of contraception or protection during their last sexual encounter. In terms of condom use over the past year, 40% (94 students) reported never using condoms, while only 3% (7 students) consistently used them. Regarding STI awareness, 15.6% (36 participants) reported having been diagnosed with an STI by a healthcare professional within the past year, although 6.5% (15 participants) did not seek medical attention (see Table 2).

**TABLE 2:** Sexual behaviours of undergraduate students at the University of Namibia, Katima Mulilo Campus (*N* = 231).

Variables	Character	*n*	%
Have you ever had sexual intercourse?	Yes	141	61.0
	No	90	39.0
Age (years) at first sexual intercourse	Below 18	51	21.1
	Above 18	90	39.0
	Never had sexual intercourse	90	39.0
During which time did you have sexual intercourse?	In primary school	20	8.7
	In secondary school	94	40.7
	After I joined the university	27	11.7
	Never had sexual intercourse	90	39.0
Reason for the first sexual intercourse	Personal desires	101	43.7
	Marriage	24	10.4
	Forced or peer pressure	12	5.2
	Never had sexual intercourse	90	39.0
With whom did you practise first sexual intercourse?	With boyfriend or girlfriend	97	42.0
	With teacher or lecturer	11	4.8
	With stranger	4	1.7
	With commercial sex worker	25	10.8
	With friends or peers	1	0.4
	Do not recall with whom	3	1.3
	Never had sexual intercourse	90	39.0
Sexual intercourse while under the influence of drugs or alcohol	Always	13	5.6
	Often	100	43.3
	Sometimes	17	7.4
	Rarely	11	4.8
	Never had sexual intercourse	90	39.0
How many sexual partners have you had in total?	1–2 people	87	37.7
	3–5 people	37	16.0
	6–9 people	17	7.4
	Never had sexual intercourse	90	39.0
Type of contraceptive used during last sexual intercourse	Birth control (hormonal)	6	2.6
	Condom	61	26.4
	Withdrawal rhythm method	44	19.0
	Other barrier methods	12	5.2
	None used	18	7.8
	Never had sexual intercourse	90	39.0
Condom usage in the last 12 months	Never used a condom	94	40.7
	25% of the time	23	10.0
	50% of the time	4	1.7
	75% of the time	13	5.6
	100% of the time	7	3.0
	Never had sexual intercourse	90	39.0
STI in the last 12 months	Yes	36	15.6
	No	180	77.9
	Never visited a health facility	15	6.5

STI, sexually transmitted infection.

Statistical analysis identified significant associations between engaging in RSBs and factors such as marital status and tobacco use.

## Discussion

This study provides valuable insights into the prevalence and determinants of RSBs among undergraduate students at a university in Namibia. Several key findings emerged, which align with trends observed in other developing countries and provide important implications for sexual health interventions.

The prevalence of sexual activity was notably high, with over 61% of participants reporting having engaged in sexual intercourse. A concerning proportion, nearly 39%, reported initiating sexual activity before the age of 18. Early initiation into sexual activity has been consistently linked with negative outcomes, such as unplanned pregnancies and STIs. Studies conducted in South Africa (Hoque 2011), Botswana (Hoque, Ntsipe & Mokgatle 2012) and Nigeria (Oharume 2020) similarly found high rates of early sexual debut, reinforcing the need for early sexual health education to delay sexual initiation and reduce associated risks.

The study revealed slight gender differences, with more females than males engaging in sexual activity. This contrasts with findings from studies in some other developing countries where males often report higher rates of sexual activity (Hoque et al. 2012; Oharume 2020). Additionally, older students, particularly those aged 24–26, were more likely to engage in sexual activity, a trend consistent with findings from other studies in African university settings, where older students tend to report higher levels of sexual activity as they near the completion of their studies (Ybarra & Mitchell 2014).

**TABLE 3:** Bivariate analysis of risky sexual behaviours across selected risk factors (*N* = 231).

Variables	Character	Yes		No		*p*
		*n*	%	*n*	%	
Gender	Female	75	32.5	53	22.9	0.396
	Male	66	28.6	37	16.0	-
Age (years)	18–20	37	16.0	19	8.2	0.387
	21–23	34	14.7	23	10.0	-
	24–26	51	22.1	38	16.5	-
	27–30	10	4.3	2	0.9	-
	31+	9	3.9	8	3.5	-
Year of study	1st year	39	16.9	20	8.7	0.418
	2nd year	37	16.0	32	13.9	-
	3rd year	30	13.0	18	7.8	-
	4th year	35	15.2	20	8.7	-
Current place of residence	On campus (hostel)	35	15.2	24	10.4	0.754
	Off-campus	106	45.9	66	28.6	-
Marital status	Single (not in a relationship)	54	23.4	31	13.4	0.049
	Single (in a relationship)	58	25.1	30	13.0	-
	Married	12	5.2	8	3.5	-
	Divorced	3	1.3	0	0.0	-
	Cohabiting	14	6.1	21	9.1	-
Where did you grow up?	Urban	46	19.9	40	17.3	0.078
	Rural	0	0.0	1	1.0	-
	Urban and rural	95	41.1	49	21.2	-
With whom did you grow up?	Biological mother and father	44	19.0	30	13.0	0.214
	Biological father	13	5.6	16	6.9	-
	Biological mother	42	18.2	23	10.0	-
	Other (extended family)	42	18.2	21	9.1	-
Area (constituency)	Kabbe South	12	5.2	5	2.2	0.125
	Kabbe North	6	2.6	9	3.9	-
	Linyanti	14	6.1	15	6.5	-
	Judea Lyamboloma	12	5.2	10	4.3	-
	Sibbinda	10	4.3	10	4.3	-
	Katima Mulilo Urban	46	19.9	19	8.2	-
	Rural Katima Mulilo	11	4.8	6	2.6	-
	Kongola	6	2.6	7	3.0	-
	Other regions in Namibia	24	10.4	9	3.9	-
Tobacco use	Smokers	39	16.9	48	20.8	0.001
	Non-smokers	102	44.2	42	18.2	-
Alcohol consumption	Drinkers	103	44.6	62	26.8	0.495
	Non-drinker	38	16.5	28	12.1	-

A critical finding was the low and inconsistent use of condoms, with 40% of participants reporting never using condoms in the past year, and only 3% consistently using them. This poses significant risks for STI transmission and unplanned pregnancies. Similar patterns have been observed in other studies in developing countries, where inconsistent condom use remains a key challenge despite awareness of contraception (Brian et al. 2016). The need for comprehensive sexual health education that promotes not only awareness but also the skills to negotiate safe sexual practices is evident.

The study also identified associations between RSBs and factors such as marital status and tobacco use. These findings suggest that social and behavioural determinants play a significant role in sexual risk-taking, as seen in similar studies conducted in other African contexts (Zango et al. 2024). Targeted interventions addressing substance abuse and promoting healthy relationships are critical to reducing RSBs among university students.

Similar findings have been observed in previous studies, where tobacco use has been linked to increased engagement in risky behaviors, including unsafe sexual practices (Amakali, Haoses-Gorases & Taukuheke 2013; Chisha & Ataguba 2018; Sreeramareddy, Pradhan & Sin 2014).

In conclusion, this study underscores the importance of comprehensive sexual health interventions tailored to the unique needs of undergraduate students in Namibia. By addressing key findings such as early sexual initiation, inconsistent condom use, and the influence of social and behavioural factors, sexual health risks among this population can be reduced. These findings align with trends observed in other developing countries, reinforcing the need for early, comprehensive sexual health education that empowers young adults to make informed decisions and engage in safer sexual practices.

### Study limitations

This study is limited by its focus on the Zambezi region, which affects the generalisability of the results to other areas in Namibia. The small sample size and use of convenience sampling may introduce bias, limiting the representativeness of the findings. The reliance on self-reported data could lead to inaccuracies because of recall and social desirability biases. Additionally, the cross-sectional design prevents establishing causality, and the lack of culturally validated tools may affect the accuracy of the results.

Additionally, while tobacco use was identified as a contributing factor to risky sexual behaviors, this study did not assess the impact of broader tobacco control policies on university students, as explored in Tam and Van Walbeek (2014).

## Conclusion

This study provides important insights into sexual health practices among university students in Namibia’s Zambezi region, revealing a significant prevalence of RSBs. Key issues identified include early sexual debut, inconsistent condom use, and limited awareness of STIs, emphasising the need for targeted interventions. To address these challenges, a multifaceted approach is essential, incorporating culturally relevant sexual health education, accessible and confidential sexual health services, and robust support systems such as counselling and peer education programmes. Empowering students to make informed sexual health decisions will enhance their overall well-being and contribute to a safer, healthier university environment.
